# Supplementary material for: A fluorescent sensor for real-time monitoring of DPP8/9 reveals crucial roles in immunity and cancer
Source: Life Sci Alliance. 2025 May 12;8(8):e202403076. doi: 10.26508/lsa.202403076 (PMC12069513; doi:10.26508/lsa.202403076)
Supplement: Supplementary file 1 [file LSA-2024-03076_SdataF1_F2_F3_F4_F5_F6_FS1_FS2_FS3_FS4_FS5_FS6_FS7_FS8_FS9.zip › Source Data/Fig1/KW_DiPAK_Fig1-1_Source-data.pdf]

C AK2-HA variant levels upon DPP9 inhibition

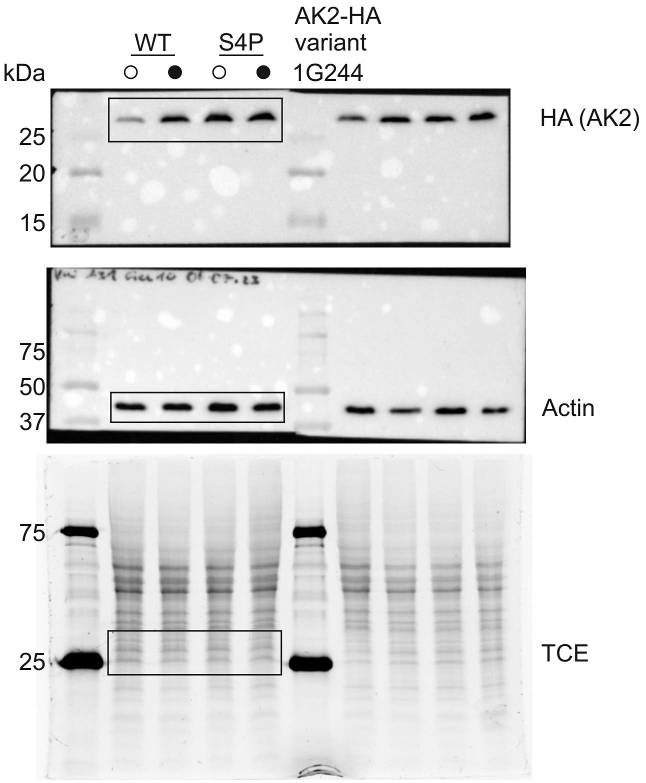

D AK2-3CS-HA variant levels upon DPP9 inhibition

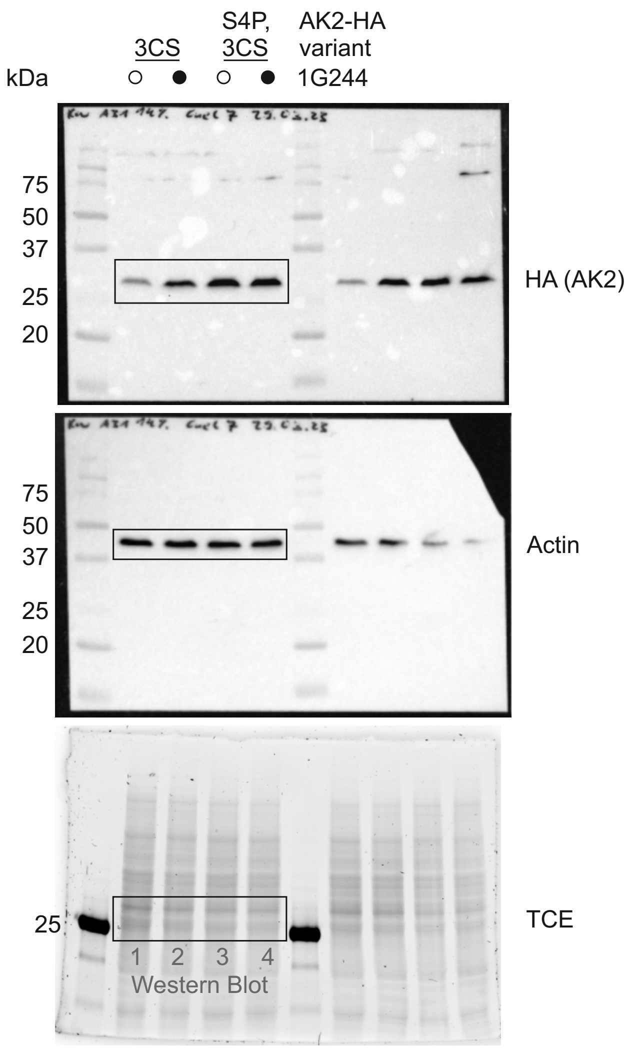

Weiss et al, Figure 1, Source Data

E AK2-3CS-HA variant levels in HEK293 WT and DPP9 KO cells

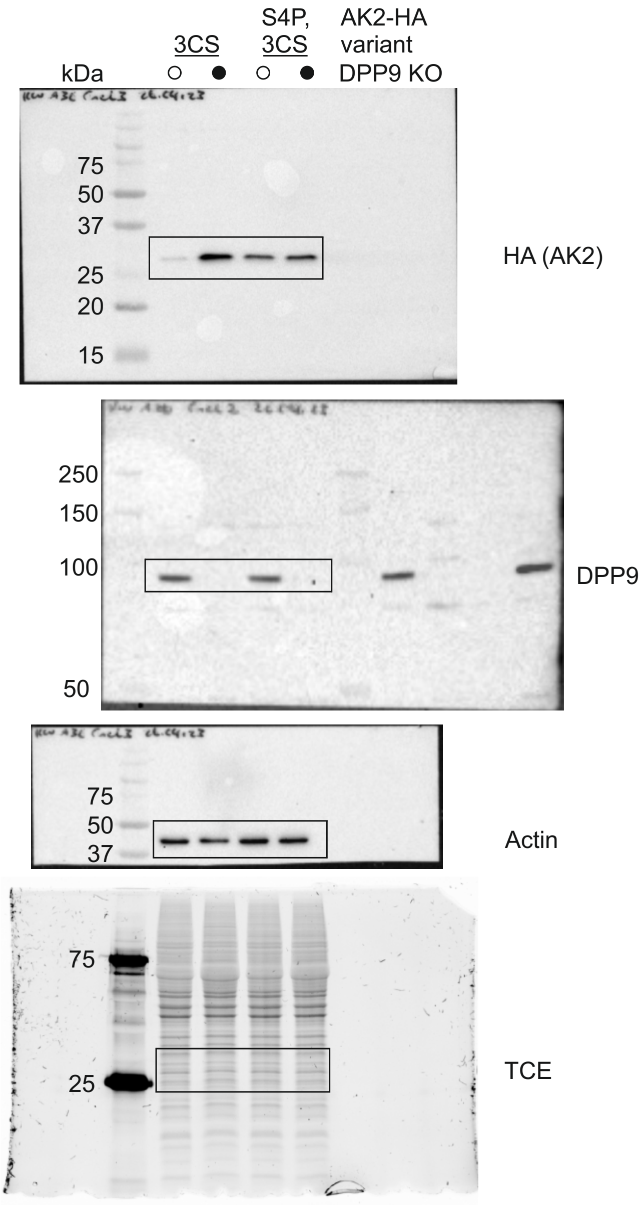

H AK2(1-15)-mEGFP levels upon DPP8/9 inhibition and DPP9 KO

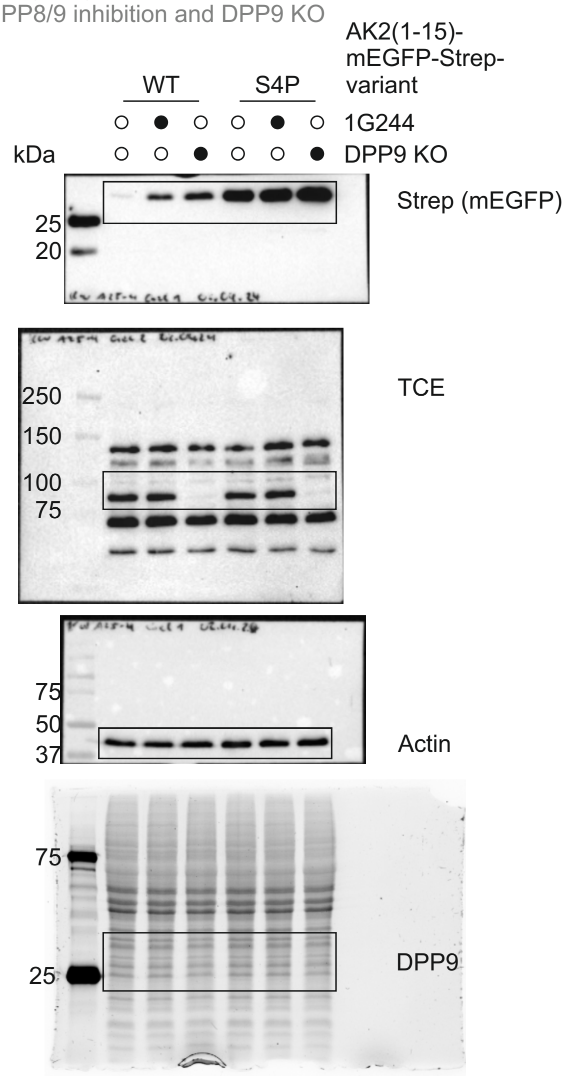

Weiss et al, Figure 1, Source Data
