# Supplementary figures and images for: A fluorescent sensor for real-time monitoring of DPP8/9 reveals crucial roles in immunity and cancer
Source: Life Sci Alliance. 2025 May 12;8(8):e202403076. doi: 10.26508/lsa.202403076 (PMC12069513; doi:10.26508/lsa.202403076)

**B** DiPAK stability in HEK/F/T WT

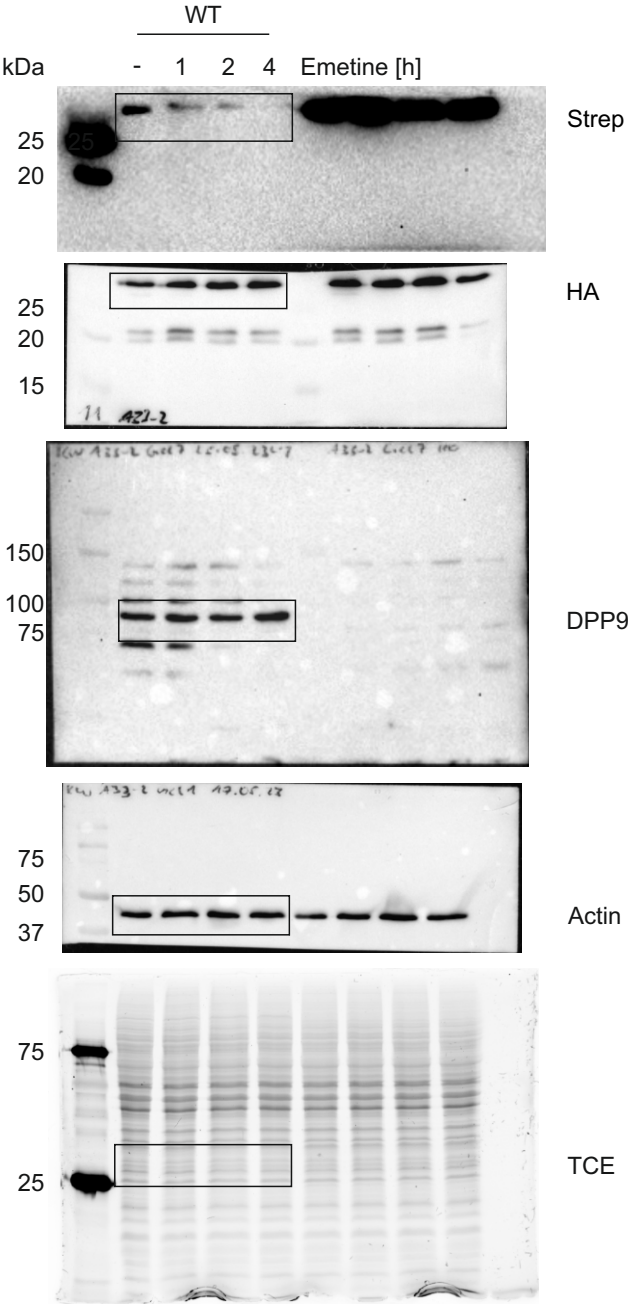

**C** DiPAK steady state Western Blot

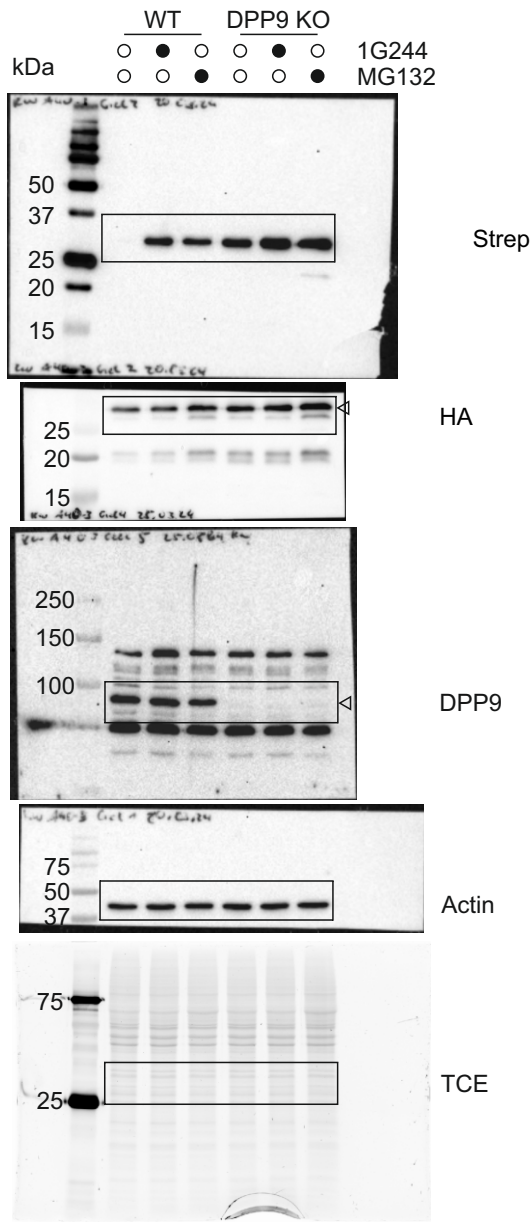

Supplement: Supplementary file 1 [file LSA-2024-03076_SdataF1_F2_F3_F4_F5_F6_FS1_FS2_FS3_FS4_FS5_FS6_FS7_FS8_FS9.zip › Source Data/Fig2/KW_DiPAK_Fig2-3_Source-data.pdf]

**B** DiPAK steady state in HeLa/F/T WT cells determined by Western Blot

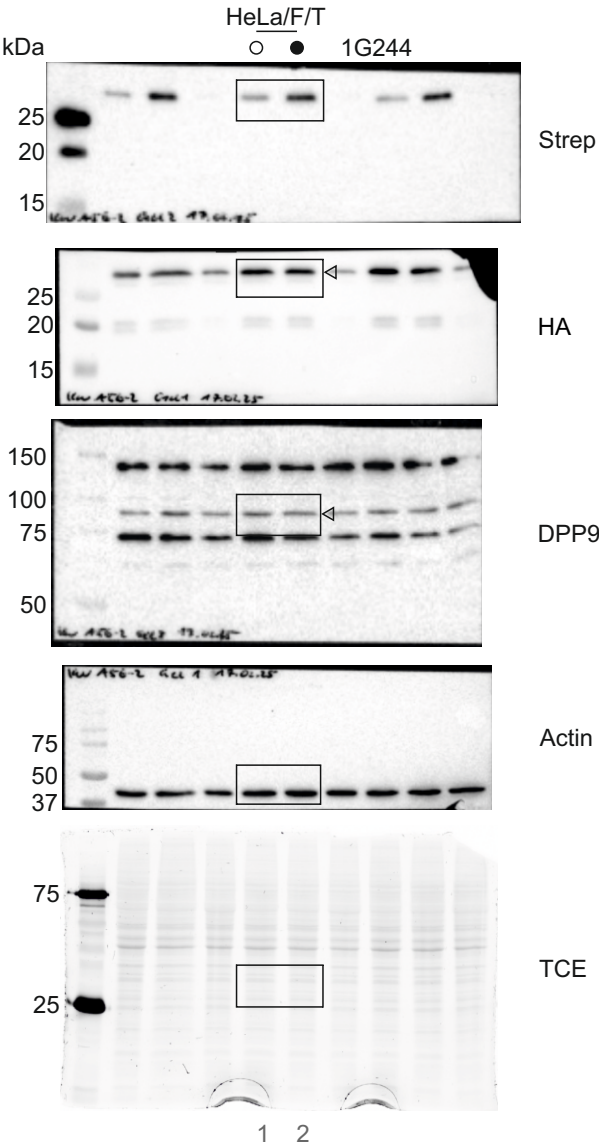

Supplement: Supplementary file 1 [file LSA-2024-03076_SdataF1_F2_F3_F4_F5_F6_FS1_FS2_FS3_FS4_FS5_FS6_FS7_FS8_FS9.zip › Source Data/FigS2/FigS2B_Source-data.pdf]

**B** WM1366 show higher DPP9 levels and lower DPP8 levels than WM3734a melanoma cells

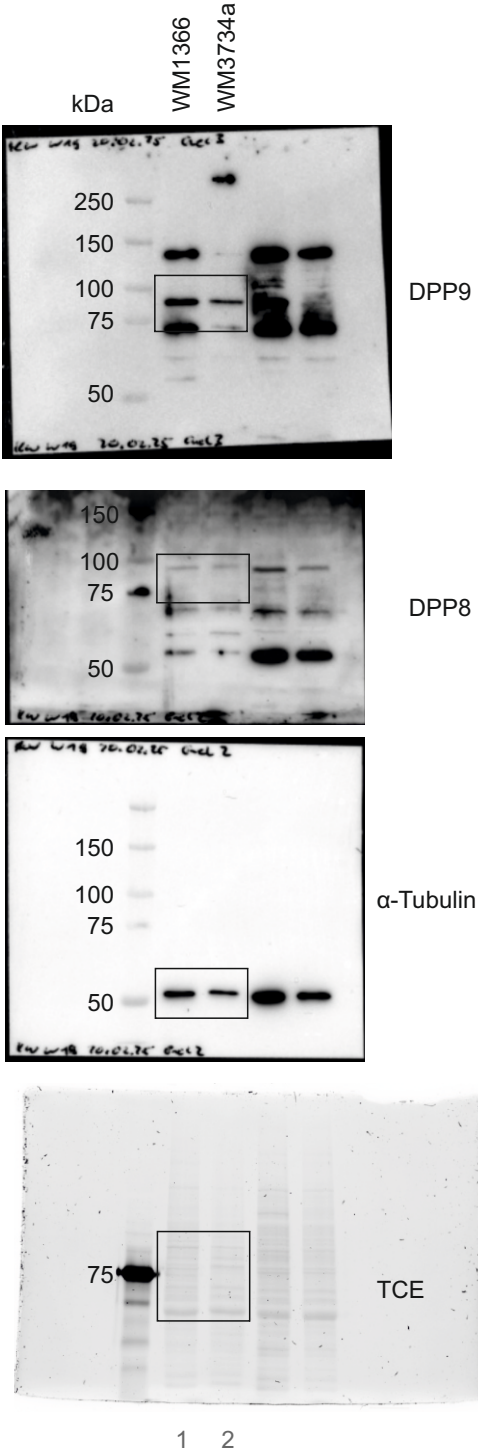

Supplement: Supplementary file 1 [file LSA-2024-03076_SdataF1_F2_F3_F4_F5_F6_FS1_FS2_FS3_FS4_FS5_FS6_FS7_FS8_FS9.zip › Source Data/FigS6/FigS6B_Source-data.pdf]
