# Supplementary material for: A fluorescent sensor for real-time monitoring of DPP8/9 reveals crucial roles in immunity and cancer
Source: Life Sci Alliance. 2025 May 12;8(8):e202403076. doi: 10.26508/lsa.202403076 (PMC12069513; doi:10.26508/lsa.202403076)
Supplement: Supplementary file 2 [file LSA-2024-03076_TableS1.docx]

Table S1. Properties of green fluorescent protein used in the “sensor unit” of DiPAK variants and red fluorescent proteins used in the “normalizer unit” of DiPAK variants. EC: Extinction coefficient, QY: Quantum yield, t_1/2_: Photostability, n.s.: not specified.

| **Fluorescent Protein** | **λ_ex_ [nm]** | **λ_em_ [nm]** | **EC [M^-1^cm^-1^]** | **QY** | **Brightness** | **pKa** | **Maturation [min]** | **Lifetime [ns]** | **t_1/2_ [s]** |
| --- | --- | --- | --- | --- | --- | --- | --- | --- | --- |
| mEGFP | 488 | 507 | 56000 | 0.6 | 33.6 | 6 | 14 | n.s. | n.s. |
| mKate2 | 588 | 633 | 62500 | 0.4 | 25 | 5.4 | 20 | 2.5 | 84 |
| mScarlet-I | 569 | 593 | 104000 | 0.54 | 56.16 | 5.4 | 36 | 3.1 | 200 |
| mCherry | 587 | 610 | 72000 | 0.22 | 15.84 | 4.5 | 15 | 1.4 | 68 |
