# Supplementary material for: A fluorescent sensor for real-time monitoring of DPP8/9 reveals crucial roles in immunity and cancer
Source: Life Sci Alliance. 2025 May 12;8(8):e202403076. doi: 10.26508/lsa.202403076 (PMC12069513; doi:10.26508/lsa.202403076)
Supplement: Supplementary file 3 [file LSA-2024-03076_TableS2.docx]

Table S2. Cell lines.

| **Cell line** | **Plasmid** | **Gene** | **Tag (C-term.)** | **Reference** |
| --- | --- | --- | --- | --- |
| HEK293 Flp-In T-REx WT | - | - | - | Invitrogen Cat# R78007 |
| HEK293 Flp-In T-REx DPP9 KO | - | - | - | Bolgi et al., 2022(1) |
| HEK293 Flp-In T-REx WT AK2-HA | pcDNA5/FRT/TO | ORF *AK2* | HA | Finger et al., 2022(2) |
| HEK293 Flp-In T-REx WT AK2-S4P-HA | pcDNA5/FRT/TO | ORF *AK2 S4P* | HA | Finger et al., 2022(2) |
| HEK293 Flp-In T-REx WT AK2-3CS-HA | pcDNA5/FRT/TO | ORF *AK2 C40,42,92S* | HA | Finger et al., 2022(2) |
| HEK293 Flp-In T-REx WT AK2-S4P,3CS-HA | pcDNA5/FRT/TO | ORF *AK2 S4P,C40S,C42S,C92S* | HA | Finger et al., 2022(2) |
| HEK293 Flp-In T-REx DPP9 KO AK2-3CS-HA | pcDNA5/FRT/TO | ORF *AK2 C40,42,92S* | HA | Finger et al., 2022(2) |
| HEK293 Flp-In T-REx DPP9 KO AK2-S4P,3CS-HA | pcDNA5/FRT/TO | ORF *AK2 S4P,C40S,C42S,C92S* | HA | Finger et al., 2022(2) |
| HEK293 Flp-In T-REx WT AK2(1-15)-mEGFP-Strep | PB-CuO-MCS-BGH-EF1-CymR-Puro | ORF *AK2 (1-15)*; mEGFP | Strep | This study |
| HEK293 Flp-In T-REx WT AK2(1-15),S4P-mEGFP-Strep | PB-CuO-MCS-BGH-EF1-CymR-Puro | ORF *AK2 (1-15),S4P*; mEGFP | Strep | This study |
| HEK293 Flp-In T-REx DPP9 KO AK2(1-15)-mEGFP-Strep | PB-CuO-MCS-BGH-EF1-CymR-Puro | ORF *AK2 (1-15)*; mEGFP | Strep | This study |
| HEK293 Flp-In T-REx DPP9 KO AK2(1-15),S4P-mEGFP-Strep | PB-CuO-MCS-BGH-EF1-CymR-Puro | ORF *AK2 (1-15),S4P*; mEGFP | Strep | This study |
| HEK293 Flp-In T-REx WT "DiPAK" AK2(1-15)-mEGFP-Strep-IRES-AK2(1-15),S4P-mKate2-HA | PB-CuO-MCS-BGH-EF1-CymR-Puro | ORF *AK2 (1-15)*; mEGFP; EMCV IRES; ORF *AK2 (1-15),S4P*; mKate2 | Strep, HA | This study |
| HEK293 Flp-In T-REx DPP9 KO "DiPAK" AK2(1-15)-mEGFP-Strep-IRES-AK2(1-15),S4P-mKate2-HA | PB-CuO-MCS-BGH-EF1-CymR-Puro | ORF *AK2 (1-15)*; mEGFP; EMCV IRES; ORF *AK2 (1-15),S4P*; mKate2 | Strep, HA | This study |
| HEK293 Flp-In T-REx DPP9 KO "DiPAK" AK2(1-15)-mEGFP-Strep-IRES-AK2(1-15),S4P-mKate2-HA; DPP9 WT | PB-CuO-MCS-BGH-EF1-CymR-Puro | ORF *AK2 (1-15)*; mEGFP; EMCV IRES; ORF *AK2 (1-15),S4P*; mKate2 | Strep, HA | This study |
|  | pcDNA5/FRT/TO | ORF *DPP9-S* | Flag |  |
| HEK293 Flp-In T-REx WT "DiPAK mKate2 only" AK2(1-15)-mEGFP-Strep-IRES-mKate2-HA | PB-CuO-MCS-BGH-EF1-CymR-Puro | ORF *AK2 (1-15)*; mEGFP; EMCV IRES; mKate2 | Strep, HA | This study |
| HEK293 Flp-In T-REx WT DHFR-myc | pcDNA5/FRT/TO | ORF *DHFR* | myc | This study |
| HeLa Flp-In T-REx WT | - | - | - | Invitrogen Cat# R71407 |
| WM1366 | - | - | - | kindly provided by Prof. Meenhard Herlyn, the Wistar Institute, Philadelphia, USA |
| WM3734a | - | - | - | kindly provided by Prof. Meenhard Herlyn, the Wistar Institute, Philadelphia, USA |
