# Supplementary material for: A fluorescent sensor for real-time monitoring of DPP8/9 reveals crucial roles in immunity and cancer
Source: Life Sci Alliance. 2025 May 12;8(8):e202403076. doi: 10.26508/lsa.202403076 (PMC12069513; doi:10.26508/lsa.202403076)
Supplement: Supplementary file 4 [file LSA-2024-03076_TableS3.docx]

Table S3. Plasmids and primers.

| **Plasmid** | **Primer (5'-3')** | **Restriction sites** | **Reference** |
| --- | --- | --- | --- |
| AK2 C40S,C42S,C92S-HA pcDNA5/FRT/TO | - | KpnI | Finger et al., 2020(2) |
|  | - | NotI |  |
| AK2 S4P,C40S,C42S,C92S-HA pcDNA5/FRT/TO | - | KpnI | Finger et al., 2020(2) |
|  | - | NotI |  |
| AK2(1-15)-mEGFP-Strep pcDNA3.1(+) | fw: gtatGCTAGCGATATCGCCGCCACCatggctcccagcgtgccagcggcagaacccgagtatcctaaaggcGGCAGCgtgagcaagggcgagg | EcoRV | This study |
|  | rv: atacGCGGCCGCTTCGAATCATTTCTC | NotI |  |
| AK2(1-15)-mEGFP-Strep pcDNA3.1(+) | fw: gtatGCTAGCGATATCGCCGCCACCatggctcccCCcgtgccagcggcagaacccgagtatcctaaaggcGGCAGCgtgagcaagggcgagg | EcoRV | This study |
|  | rv: atacGCGGCCGCTTCGAATCATTTCTC | NotI |  |
| "DiPAK" AK2(1-15)-WT-mEGFP-Strep_IRES_AK2(1-15)-S4P-mKate2-HA PB-CuO-MCS-BGH-EF1-CymR-Puro | - | NheI | This study |
|  | - | NotI |  |
| "DiPAK mKate2 only" AK2(1-15)-WT-mEGFP-Strep_IRES_mKate2-HA PB-CuO-MCS-BGH-EF1-CymR-Puro | - | NheI | This study |
|  | - | NotI |  |
| "DiPAK" AK2(1-15)-WT-mEGFP-Strep_IRES_AK2(1-15)-S4P-mKate2-HA pcDNA3.1(+) | - | EcorRV | This study |
|  | - | NotI |  |
| "DiPAK mScarlet-I" AK2(1-15)-WT-mEGFP-Strep_IRES_AK2(1-15)-S4P-mScarlet-I-HA pcDNA3.1(+) | - | EcorRV | This study |
|  | - | NotI |  |
| "DiPAK mCherry" AK2(1-15)-WT-mEGFP-Strep_IRES_AK2(1-15)-S4P-mCherry-HA pcDNA3.1(+) | - | EcorRV | This study |
|  | - | NotI |  |
| "DiPAK" AK2(1-15)-WT-mEGFP-Strep_IRES_AK2(1-15)-S4P-mKate2-HA pCru5 | fw: accatcctctagactgccggatcAGCGATATCGCCGCCACCatgg | Gibson assembly | This study |
|  | rv: ctggagactaaataaaatcttttattttatAGCGGCCGCTCAtgcataatcagg | Gibson assembly |  |
| DPP9-S-Flag | - | KpnI | Bolgi et al., 2022(1) |
|  | - | BamHI |  |
| DHFR-myc | fw: gtatGGTACCGCTAGCGCCGCCACCatggttcgaccattgaactgc | KpnI | This study |
|  | rv: gtatGCGGCCGCttaCAGGTCCTCCTCGCTGATCAGCTTCTGCTCgtctttcttctcgtagac | NotI |  |
