# Supplementary material for: A fluorescent sensor for real-time monitoring of DPP8/9 reveals crucial roles in immunity and cancer
Source: Life Sci Alliance. 2025 May 12;8(8):e202403076. doi: 10.26508/lsa.202403076 (PMC12069513; doi:10.26508/lsa.202403076)
Supplement: Supplementary file 5 [file LSA-2024-03076_TableS4.docx]

Table S4. Antibodies.

| **Antibody** | **Company** | **Identifier** |
| --- | --- | --- |
| Rabbit polyclonal anti HA | Sigma-Aldrich | Cat# SAB4300603; RRID:AB_10620829 |
| Mouse monoclonal anti Actin | ThermoFisher Scientific | Cat# #MA5-11869 |
| Rabbit polyclonal anti DPP9 | abcam | Cat# ab42080 |
| Rabbit polyclonal anti DPP8 | Proteintech | Cat# 12752-1-AP |
| Mouse monoclonal anti α-Tubulin | Sigma-Aldrich | Cat# T6074 |
| Mouse monoclonal anti Strep | Qiagen | Cat# 34850 |
| Goat anti-Mouse lgG (H&L), HRP Conjugate | ImmunoReagents | Cat# GtxMu-003-DHRPX |
| Goat anti-Rabbit lgG (H&L), HRP Conjugate | ImmunoReagents | Cat# GtxRb-003-DHRPX |
| Rat monoclonal anti TACI/CD267 (APC, clone eBio8F10-3) | eBioscience | Cat# 17-5942-82, RRID:AB_842758 |
| Rat monoclonal anti CD19 (APCFire750, clone 6D5) | Biolegend | Cat# 115558, RRID:AB_2572120 |
| Rat monoclonal anti B220/CD45R (PerCP Cy5.5, clone Ra3-6b) | eBioscience | Cat# 45-0452-80, RRID:AB_906234 |
| Rat monoclonal anti CD138 (PECy7, clone 281-2) | Biolegend | Cat# 142513, RRID:AB_2562197 |
| Rat monoclonal anti GL-7 (FITC, clone GL-7) | BD Biosciences | Cat# 553666, RRID:AB_394981 |
| Goat polyclonal anti IgD rPE | SouthernBiotech | Cat# 2032-09 |
| Mouse monoclonal anti CD20 APC-Cy7 | BioLegend | Cat# 302314 |
| Human BD Fc Block™ | BD Bioscience | Cat# 564219 |
| Mouse monoclonal anti CD4 APC | BioLegend | Cat # 317416 |
| Rabbit polyclonal CD27 FITC | Dako | Cat# F7178 |
| Mouse monoclonal CD38 PE Cy7 | BioLegend | Cat# 356608 |
