# Supplementary material for: A fluorescent sensor for real-time monitoring of DPP8/9 reveals crucial roles in immunity and cancer
Source: Life Sci Alliance. 2025 May 12;8(8):e202403076. doi: 10.26508/lsa.202403076 (PMC12069513; doi:10.26508/lsa.202403076)
Supplement: Supplementary file 6 [file LSA-2024-03076_TableS5.docx]

Table S5. Chemicals and further tools.

| **Chemical/ reagent** | **Company** | **Identifier** |
| --- | --- | --- |
| 1G244 | MedChemExpress | Cat# HY-116304 |
| Sitagliptin | MedChemExpress | Cat# HY-13749 |
| KYP-2047 | MedChemExpress | Cat# HY-100475 |
| Emetine dihydrochloride hydrate | Sigma-Aldrich | Cat# 7083-71-8 |
| ROTI®Quant universal | Carl Roth | Cat# 0120.1 |
| MG132 | Sigma-Aldrich | Cat# C2211 |
| GP-AMC | Bachem | Cat# 4016648.0250 |
| DAPI [4',6-Diamidino-2-phenylindol Dihydrochlorid] | Roth | Cat# 6843.2 |
| PrestoBlue Cell viability reagent | Thermo Fisher Scientific | Cat# A13262 |
| Apotracker^TM^ Green | Biolegend | Cat# 427403 |
| eBioScience^TM^ Fixable Viability Dye eFluor^TM^ 780 | Invitrogen | Cat# 65-0865-14 |
