# Supplementary material for: A fluorescent sensor for real-time monitoring of DPP8/9 reveals crucial roles in immunity and cancer
Source: Life Sci Alliance. 2025 May 12;8(8):e202403076. doi: 10.26508/lsa.202403076 (PMC12069513; doi:10.26508/lsa.202403076)
Supplement: Supplementary file 7 [file LSA-2024-03076_TableS6.docx]

Table S6. Experimental models.

| **Model (organism/strain)** | **Identifier** | **Reference** |
| --- | --- | --- |
| R26-K320E-TWINKLE loxP+/- | - | Baris et al., 2015(3) |
| Tg(Fcer2a-cre)5Mbu | - | Kwon et al., 2008(4) |
| SLC2A1 loxP+/- | - | Young et al., 2011(5) |
| HEK293 Flp-In T-REx cell lines | - | see Table S1 |
| Human melanoma cell lines | - | see Table S1 |
