# Supplementary material for: A fluorescent sensor for real-time monitoring of DPP8/9 reveals crucial roles in immunity and cancer
Source: Life Sci Alliance. 2025 May 12;8(8):e202403076. doi: 10.26508/lsa.202403076 (PMC12069513; doi:10.26508/lsa.202403076)
Supplement: Supplementary file 8 [file LSA-2024-03076_TableS7.docx]

Table S7. Software and Algorithms.

| **Software/ Algorithm** | **Source** | **Reference** |
| --- | --- | --- |
| Image Lab 5.2 | BioRad | https://www.bio-rad.com/ |
| AlphaFold2 | DeepMind | Jumper et al, 2021(6) |
| Pymol | Schrödinger | https://www.pymol.org |
| Redox ratio analysis "RRA" | Mark Fricker | Fricker, 2016(7) |
| R | R Foundation | https://www.r-project.org/ |
| Rstudio IDE | RStudio Team | https://www.rstudio.com/ |
| ZEN | Carl Zeiss Microscopy | https://www.zeiss.com/ |
| OMERO.insight | Open Microscopy Environment | https://www.openmicroscopy.org/ |
| BioTek Gen5 | Agilent | https://www.agilent.com/ |
| FlowJo V7.6.3 software | Treestar | https://www.flowjo.com/ |
| Seahorse Wave Desktop Software | Agilent | https://www.agilent.com/en/product/cell-analysis/real-time-cell-metabolic-analysis/xf-software/seahorse-wave-desktop-software-740897 |
| Kaluza | Beckman Coulter | https://www.beckman.de/flow-cytometry/software/kaluza |
| GraphPad Prism 9 | Graphpad | https://www.graphpad.com/scientific-software/prism/ |
